# Hirsutane Sesquiterpenes from Cultures of the Basidiomycete *Marasmiellus* sp. BCC 22389

**DOI:** 10.1007/s13659-016-0105-7

**Published:** 2016-08-26

**Authors:** Masahiko Isaka, Somporn Palasarn, Malipan Sappan, Sumalee Supothina, Thitiya Boonpratuang

**Affiliations:** National Center for Genetic Engineering and Biotechnology (BIOTEC), 113 Thailand Science Park, Phaholyothin Road, KlongLuang, Pathumthani, 12120 Thailand

**Keywords:** *Marasmiellus*, Basidiomycete, Hirsutane sesquiterpenoid

## Abstract

**Abstract:**

Two new hirsutane sesquiterpenes, marasmiellins A (**1**) and B (**2**), were isolated from cultures of the basidiomycete *Marasmiellus* sp. BCC 22389. The structures were elucidated on the basis of NMR spectroscopic and mass spectrometry data. The absolute configuration of marasmiellin B was determined by application of the modified Mosher’s method.

**Graphical Abstract:**

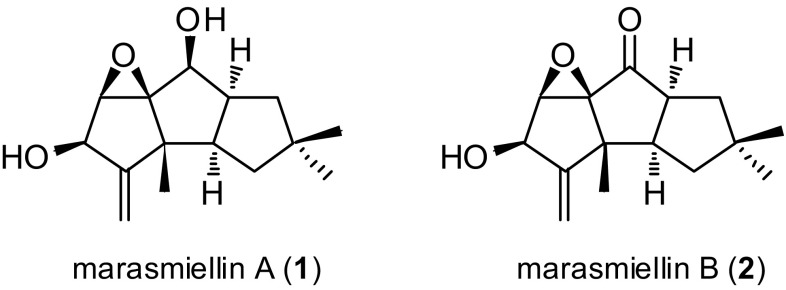

**Electronic supplementary material:**

The online version of this article (doi:10.1007/s13659-016-0105-7) contains supplementary material, which is available to authorized users.

## Introduction

**Fig. 1:**
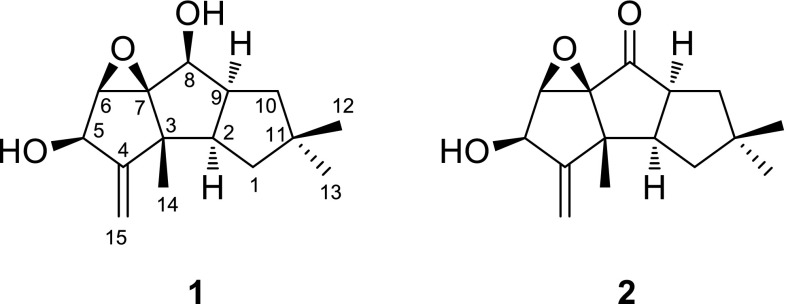
Structures of marasmiellins A (**1**) and B (**2**)

Mushrooms have been valued as potent sources of bioactive compounds [1, 2]. While some medicinal species have been well investigated, many species remain chemically unexplored or poorly studied. *Marasmiellus* is a relatively large genus composed of more than 400 described species/subspecies; however, there have been only a few reports on the chemical constituents from this genus: *cis*-caryophillane sesquiterpenes from fermentation broth of *Marasmiellus troyanus* [3], picolinic acid derivatives (CJ-14,877 and CJ-14,897; cytokine production inhibitors) from *Marasmiellus* sp. CL21624 [4], and benzoxepine derivatives from *M. ramealis* [5, 6]. As part of our research program on utilization of fungal sources in Thailand, bioassay and chemical profile-based screenings were performed in an effort to discover novel bioactive compounds with diverse chemical structures. In particular, we have recently been focusing on basidiomycetes as diverse sources of bioactive terpenoids [7–9]. Reported herein are the results of the chemical investigation of *Marasmiellus* sp. BCC 22389. Although an extract from cell cultures from this fungus were inactive in a panel of biological assays, it displayed a unique and complex ^1^H NMR profile, demonstrating the occurrence of terpenoids. Scale-up fermentation and chemical studies of BCC 22389 led to the isolation and characterization of two new hirsutane-type sesquiterpenes, marasmiellins A (**1**) and B (**2**) (Fig. 1).

## Results and Discussion

**Fig. 2:**
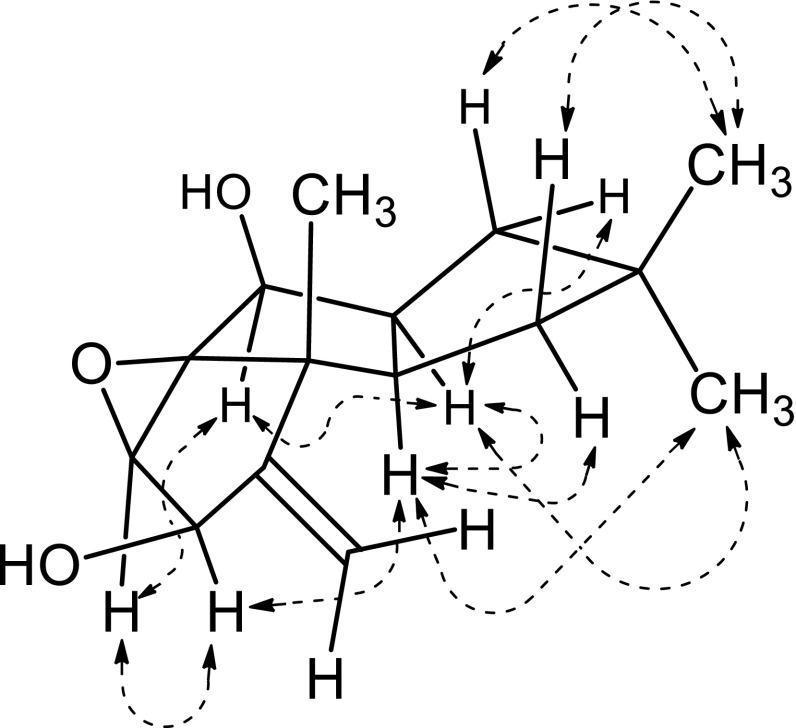
Key NOESY correlations for **1**

The molecular formula of marasmiellin A (**1**) was determined by HRESIMS as C_15_H_22_O_3_. The ^13^C NMR, DEPT135 and HMQC spectroscopic data indicated the presence of 15 carbons categorized as an exomethylene group (*δ*_C_ 160.4, qC; *δ*_C_ 112.0, CH_2_), an oxygenated quaternary carbon (*δ*_C_ 77.3), three oxymethines (*δ*_C_ 74.2, 70.7 and 63.8), two *sp* ^3^ quaternary carbons, two methine, two methylene, and three methyl groups (Table 1). The planar structure was deduced from ^1^H-^1^H COSY and HMBC data to be a typical hirsutane sesquiterpene. The COSY data indicated the linkage of C-1–C-2–C-9–C-10 and connection of an oxymethine (C-8, *δ*_C_ 70.7; H-8, *δ*_H_ 4.02) to C-9. The presence of geminal dimethyl groups (CH_3_-12 and CH_3_-13) was revealed by the HMBC correlations of these methyl protons to the other methyl carbon to each other (H_3_-12 to C-13, and H_3_-13 to C-12), and the correlations from both methyl protons (H_3_-12 and H_3_-13) to attached quaternary carbon (C-11) and two methylene carbons (C-1 and C-10). These data revealed the presence of a five-membered ring composed of C-1, C-2, C-9, C-10, and C-11. HMBC correlations from H-1 (*δ*_H_ 1.50), H-2, and H-9 to a quaternary carbon at *δ*_C_ 48.6 (C-3) indicated the C-2–C-3 bond. Methyl protons at *δ*_H_ 1.22 (H_3_-14) showed intense HMBC correlations to C-2, C-3, an exomethylene quaternary carbon (C-4), and an oxygenated quaternary carbon (C-7), which demonstrated the attachment of CH_3_-14 to C-3. HMBC correlations from H-8 to C-9, C-2, C-3, C-7, and an oxygenated methine carbon (*δ*_C_ 63.8, C-6) revealed the C-7–C-8 bond to form the central five-membered ring (C-2, C-3, C-7, C-8, and C-9), and connection of C-6 to C-7. COSY correlation of H-6 with another oxymethine proton at *δ*_H_ 4.61 (H-5), and HMBC correlations from exomethylene protons (H_2_-15) to C-3, C-4, and C-5 requested the terminal five-membered ring structure (C-3, C-4, C-5, C-6, C-7). Finally, presence of an epoxide was a requirement from the molecular formula (HRMS). The location of the epoxide was assigned to be C-6/C-7 on the basis of the chemical shifts of H-5, C-5, and C-6, therefore, a hirsutane skeleton was established for **1**. The relative configuration of **1** was determined on the basis of the NOESY correlations (Fig. 2). The round NOESY correlation network of H-5/H-6, H-6/H-8, H-8/H-9, and H-9/H-2 indicated that all these protons oriented on the same side (α) of the tricyclic ring, and these data also requested the *cis*/*cis* ring junctions and β-orientation of the epoxide and CH_3_-14. The assignments of protons for H_α_-1/H_β_-1, H_α_-10/H_β_-10, and H_3_-12/H_3_-13 were also established on the basis of the NOESY correlations.

**Table 1:** NMR spectroscopic data for **1** and **2** (CDCl_3_, 400 MHz for ^1^H NMR, 100 MHz for ^13^C NMR)

Position	Marasmiellin A (**1**)			Marasmiellin B (**2**)		
	*δ*_C_, multi	*δ*_H_, multi (*J* in Hz)	HMBC	*δ*_C_, multi	*δ*_H_, multi (*J* in Hz)	HMBC
1	40.5, CH_2_	α 1.45, m; β 1.50, m	2, 3, 9, 10, 11, 13	40.3, CH_2_	α 1.68, m; β 1.70, m	2, 3, 10
2	49.3, CH	2.31, m	1, 3, 4, 9	44.7, CH	2.65, m	1, 3, 4, 10, 12
3	48.6, qC			47.7, qC		
4	160.4, qC			158.1, qC		
5	74.2, CH	4.61, br d (8.4)		74.4, CH	4.76, br d (8.8)	4
5-O*H*		1.76, br d (8.4)			1.79, br d (8.8)	
6	63.8, CH	3.57, d (2.0)	4, 5	65.5, CH	3.83, d (1.9)	4, 5, 8
7	77.3, qC			75.0, qC		
8	70.7, CH	4.02, d (6.7)	2, 3, 6, 7	210.0, qC		
9	45.7, CH	2.84, m	1, 2, 3, 10	50.5, CH	3.09, ddd (12.0, 11.1, 8.6)	3, 8, 10
10	38.9, CH_2_	α 1.38, m	1, 2, 11, 13	42.8, CH_2_	α 1.83, dd (12.4, 8.6)	1, 2, 13
		β 1.74, t (11.7)	8, 9, 11, 12, 13		β 1.74, dd (12.4, 12.0)	8, 9, 11
11	41.9, qC			42.3, qC		
12	29.2, CH_3_	1.14, s	1, 10, 11, 13	28.6, CH_3_	1.15, s	1, 10, 11, 13
13	27.6, CH_2_	0.94, s	1, 10, 11, 12	28.2, CH_3_	0.96, s	1, 10, 11, 12
14	18.8, CH_3_	1.22, s	2, 3, 4, 7	18.6, CH_3_	1.14, s	2, 3, 4, 7
15	112.0, CH_2_	5.27, d (2.1); 5.03, d (2.1)	3, 4, 5	113.7, CH_2_	5.39, d (2.1); 5.14, d (2.1)	3, 4, 5

**Fig. 3:**
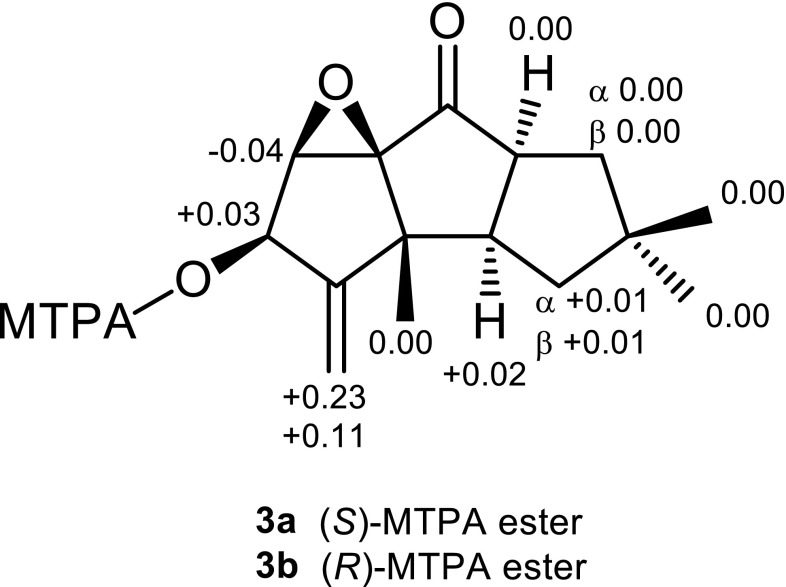
Δ*δ*-Values (*δ*_*S*_–*δ*_*R*_) of the Mosher esters **3a** and **3b**

The molecular formula of marasmiellin B (**2**) was determined by HRESIMS as C_15_H_20_O_3_. The ^1^H and ^13^C NMR spectroscopic data were similar to those of **1**. A remarkable difference was the presence of a ketone (*δ*_C_ 210.0) replacing the oxymethine (C-8) in **1**. HMBC correlations from H-9 and H_α_-10 (*δ*_H_ 1.83) to the ketone carbon indicated the location. Detailed analyses of the NOESY spectrum confirmed that its relative configuration was identical to **1**. Consequently, marasmiellin B (**2**) was identified as the C-8 ketone variant of **1**. The absolute configuration of **2** was determined by application of the modified Mosher’s method [10]. The Δ*δ* values of the (*S*)- and (*R*)-MTPA esters **3a** and **3b**, respectively, indicated the 5*R* configuration (Fig. 3).

Marasmiellins are hirstane-type sesquiterpenes structurally close related to coriolins [11]. Although this class of secondary metabolites are common in basidiomycetes, this is the first report of the isolation from *Marasmiellus* genus. Compounds **1** and **2** were inactive in the cytotoxicity assays against cancer cell-lines (KB, MCF-7, and NCI-H187) [12] at a concentration of 50 μg/mL. They were also inactive in assays for antitubercular (*Mycobacterium tuberculosis* H37Ra) and antimalarial (*Plasmodium falciparum* K1) activities.

## Experimental

### General Experimental Procedures

Melting points were measured with an Electrothermal IA9100 digital melting point apparatus. Optical rotations were measured with a JASCO P-1030 digital polarimeter. UV spectra were recorded on an Analytik Jena SPEKOL 1200 spectrophotometer. IR spectra were taken on a Bruker ALPHA spectrometer. NMR spectra were recorded on a Bruker DRX400 spectrometer. ESITOF mass spectra were measured with a Bruker micrOTOF mass spectrometer.

### Fungal Material

The fungus used in this study was collected on an unidentified decayed twig in Sakarat Research Unit, Chachoengsao province, Thailand. The natural mushroom specimen was deposited in the BIOTEC Bangkok Herbarium as BBH 16982. The living culture was deposited in the BIOTEC Culture Collection on July 27, 2006, as BCC 22389. On the basis of the morphology of the mushroom specimen and the ITS rDNA sequence data (GenBank accession number: KT800055) this fungus was identified as the genus *Marasmiellus* of the family Marasmiaceae, but it was not assignable to the species level.

### Fermentation, Extraction, and Isolation

The fungus BCC 22389 was fermented in a 1000 mL Erlenmeyer flask containing 250 mL of malt extract broth (MEB; malt extract 6.0 g/L, yeast extract 1.2 g/L, maltose 1.8 g/L, dextrose 6.0 g/L) at 25 °C for 38 days under static conditions. The cultures were filtered to separate broth and mycelia (residue). The broth was extracted with EtOAc (3 × 50 mL) and concentrated under reduced pressure to obtain a brown gum (broth extract, 34 mg). The wet mycelia were macerated in MeOH (200 mL, rt, 2 days) and filtered. Hexanes (150 mL) and H_2_O (50 mL) were added to the filtrate, and the layers were separated. The H_2_O/MeOH (bottom) layer was partially concentrated by evaporation, and the residue was extracted with EtOAc (200 mL). The EtOAc layer was concentrated under reduced pressure to obtain a brown gum (mycelial extract, 26 mg). The broth extract was passed through a column on Sephadex LH-20 (2.8 × 50 cm) and eluted with MeOH to obtain three pooled fractions. Fraction 2 (21 mg) was subjected to column chromatography (CC) on silica gel (1.8 × 15 cm, MeOH/CH_2_Cl_2_, step gradient elution from 0:100 to 20:80) to furnish **2** (4.1 mg) and **1** (4.0 mg). The mycelial extract was also fractionated using the similar chromatographic protocols to give **2** (1.5 mg) and **1** (1.1 mg).

Marasmiellin A (**1**): colorless solid; mp 140–141°C; $[\alpha ]^{ 2 6}_{\text{D}}$+53 (*c* 0.12, MeOH); UV (MeOH) λ_max_ (log *ε*): 210 (3.21) nm; IR (ATR) ν_max_ 3218, 2952, 1742, 1071, 1058, 924, 774 cm^−1^; for ^1^H NMR (400 MHz, CDCl_3_) and ^13^C NMR (100 MHz, CDCl_3_) data, see Table 1; HRESIMS: *m*/*z* 273.1466 (calcd for C_15_H_22_O_3_Na [M+Na]^+^, 273.1461).

Marasmiellin B (**2**): colorless solid; mp 122–123°C; $[\alpha ]^{ 2 6}_{\text{D}}$+43 (*c* 0.09, MeOH); UV (MeOH) λ_max_ (log *ε*): 215 (3.32) nm; IR (ATR)ν_max_ 3407, 2952, 1088, 940, 773 cm^−1^; for ^1^H NMR (400 MHz, CDCl_3_) and ^13^C NMR (100 MHz, CDCl_3_) data, see Table 1; HRESIMS *m*/*z* 271.1306 (calcd for C_15_H_20_O_3_Na [M+Na]^+^, 271.1305).

### Synthesis of Mosher Esters **3a** and **3b**

Compound **2** (0.5 mg) was treated with (-)-(*R*)-MTPA-Cl (15 μL) in pyridine (0.2 mL) at room temperature for 16 h. The mixture was diluted with EtOAc and washed with H_2_O and 1 M NaHCO_3_. The organic layer was dried over anhydrous MgSO_4_ and concentrated in vacuo to give a mixture containing a (*S*)-MTPA ester derivative **3a**: ^1^H NMR (400 MHz, CDCl_3_) *δ* 7.57–7.41 (5H, m, phenyl of MTPA), 5.90 (1H, br s, H-5), 5.24 (1H, s, H_a_-15), 5.18 (1H, s, H_b_-15), 4.05 (1H, s, H-6), 3.55 (3H, br s, –OC*H*_3_ of MTPA), 3.14 (1H, m, H-9), 2.76 (1H, m, H-2), 1.85 (1H, m, H_α_-10), 1.73 (1H, m, H_β_-1), 1.70 (1H, m, H_α_-1), 1.45 (1H, t, *J* = 12.1 Hz, H_β_-10), 1.15 (6H, s, H-12 and H-14), 0.98 (3H, s, H-13). Similarly, (*R*)-MTPA ester derivative **3b** was prepared from **2** and (+)-(*S*)-MTPA-Cl: ^1^H NMR (400 MHz, CDCl_3_) *δ* 7.58–7.40 (5H, m, phenyl of MTPA), 5.87 (1H, s, H-5), 5.09 (1H, s, H_a_-15), 5.01 (1H, s, H_b_-15), 4.09 (1H, s, H-6), 3.59 (3H, br s, –OC*H*_3_ of MTPA), 3.14 (1H, m, H-9), 2.74 (1H, m, H-2), 1.85 (1H, dd, *J* = 12.7, 8.1 Hz, H_α_-10), 1.72 (1H, m, H_β_-1), 1.71 (1H, m, H_α_-1), 1.45 (1H, t, *J* = 12.4 Hz, H_β_-10), 1.15 (6H, s, H-12 and H-14), 0.98 (3H, s, H-13).

## Electronic supplementary material

Below is the link to the electronic supplementary material.

Supplementary material 1 (PDF 3174 kb)
