# Supplementary material for: Hirsutane Sesquiterpenes from Cultures of the Basidiomycete Marasmiellus sp. BCC 22389
Source: Nat Prod Bioprospect. 2016 Aug 26;6(5):257–60. doi: 10.1007/s13659-016-0105-7 (PMC5080207; doi:10.1007/s13659-016-0105-7)

Supporting Information for:

Hirsutane Sesquiterpenes from Cultures of the Basidiomycete *Marasmiellus* sp. BCC 22389

Masahiko Isaka*, Somporn Palasarn, Malipan Sappan, Sumalee Supothina and Thitiya Boonpratuang

Contents

NMR spectra of marasmiellins A (**1**) and B (**2**)

S1	^{1}H NMR spectrum of **1** ($CDCl_3$, 400 MHz)
S2	^{13}C NMR spectrum of **1** ($CDCl_3$, 100 MHz)
S3	DEPT135 spectrum of **1** ($CDCl_3$, 100 MHz)
S4	COSY spectrum of **1** ($CDCl_3$, 400 MHz)
S5	NOESY spectrum of **1** ($CDCl_3$, 400 MHz)
S6	HMQC spectrum of **1** ($CDCl_3$, 400 MHz)
S7	HMBC spectrum of **1** ($CDCl_3$, 400 MHz)
S8	^{1}H NMR spectrum of **2** ($CDCl_3$, 400 MHz)
S9	^{13}C NMR spectrum of **2** ($CDCl_3$, 100 MHz)
S10	DEPT135 spectrum of **2** $CDCl_3$, 100 MHz)
S11	COSY spectrum of **2** ($CDCl_3$, 400 MHz)
S12	NOESY spectrum of **2** ($CDCl_3$, 400 MHz)
S13	HMQC spectrum of **2** ($CDCl_3$, 400 MHz)
S14	HMBC spectrum of **2** ($CDCl_3$, 400 MHz)

S1 ^{1}H NMR spectrum of **1** ($CDCl_3$, 400 MHz)

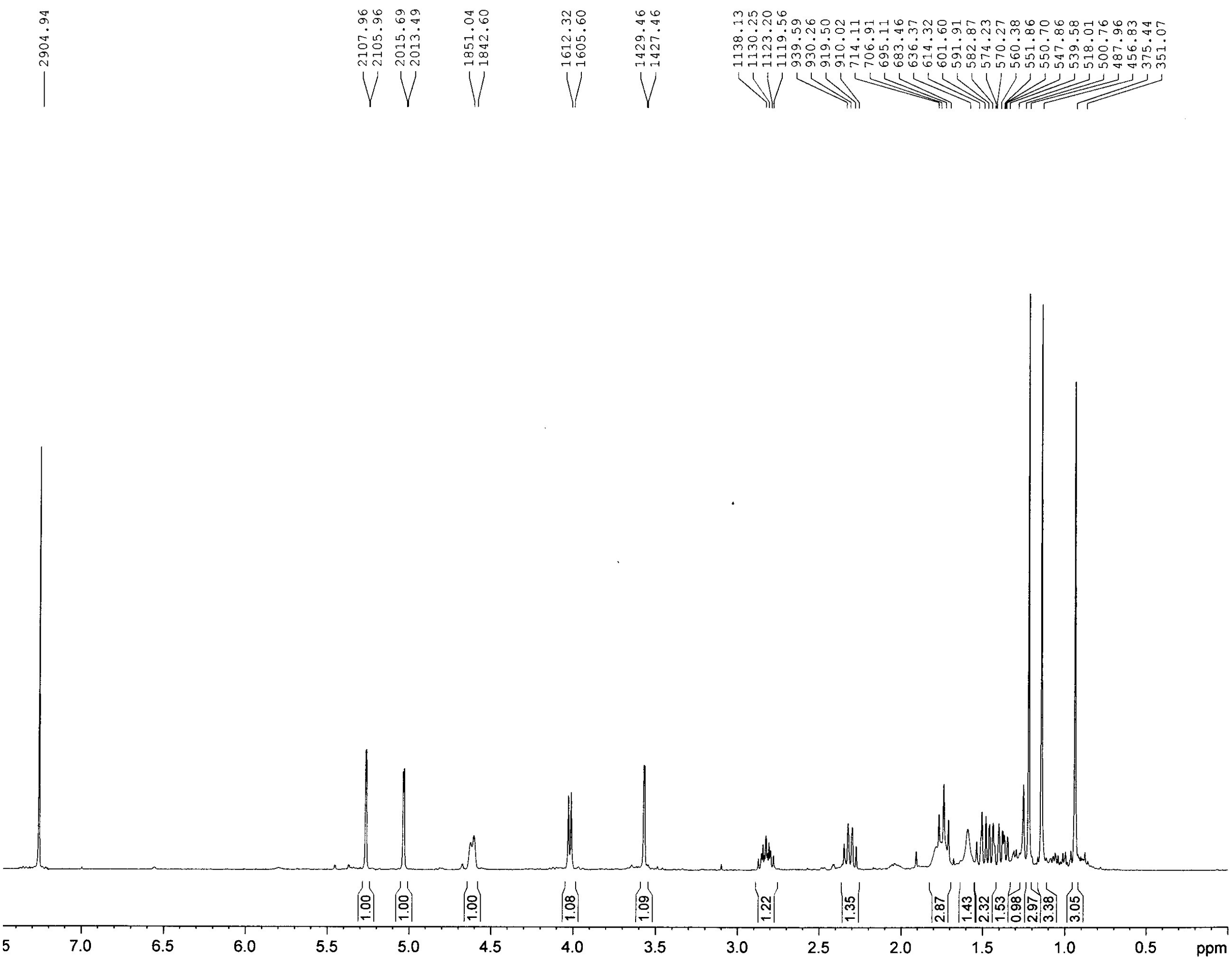

S2 ^{13}C NMR spectrum of **1** ($CDCl_3$, 100 MHz)

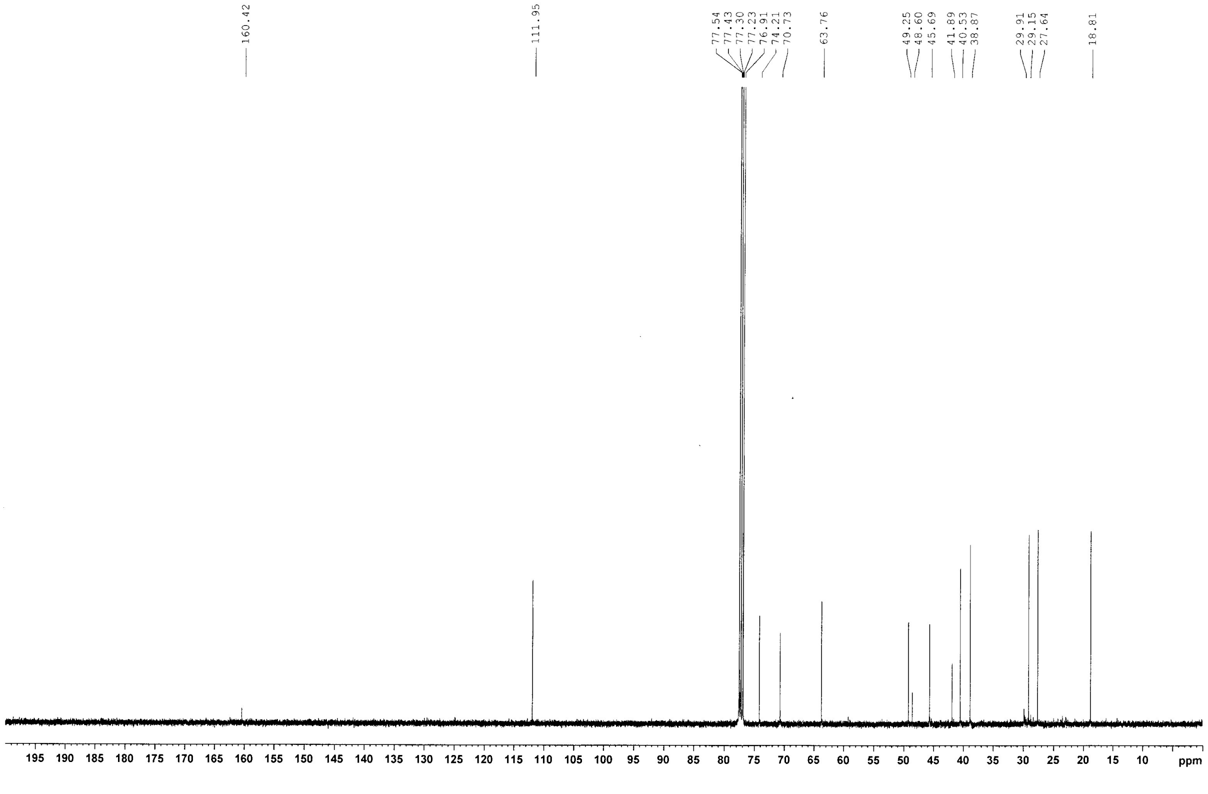

S3 DEPT135 spectrum of **1** ($CDCl_3$, 100 MHz)

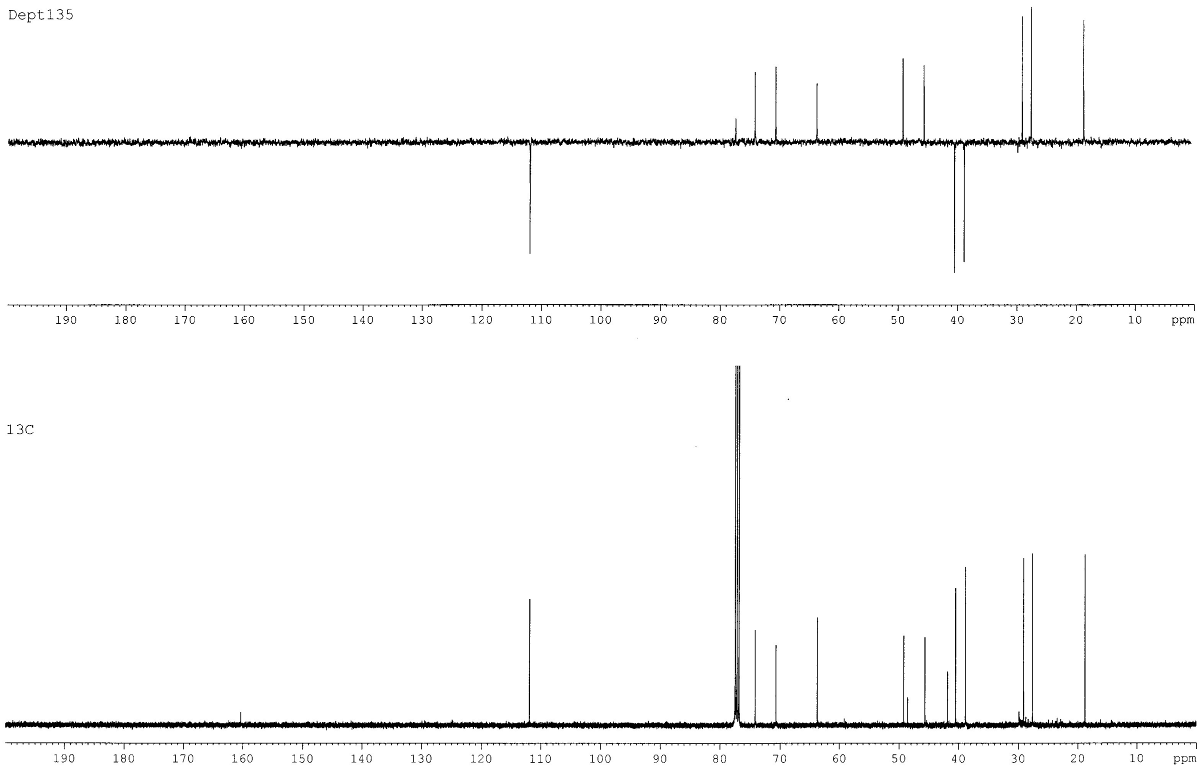

S4 COSY spectrum of **1** ($CDCl_3$, 400 MHz)

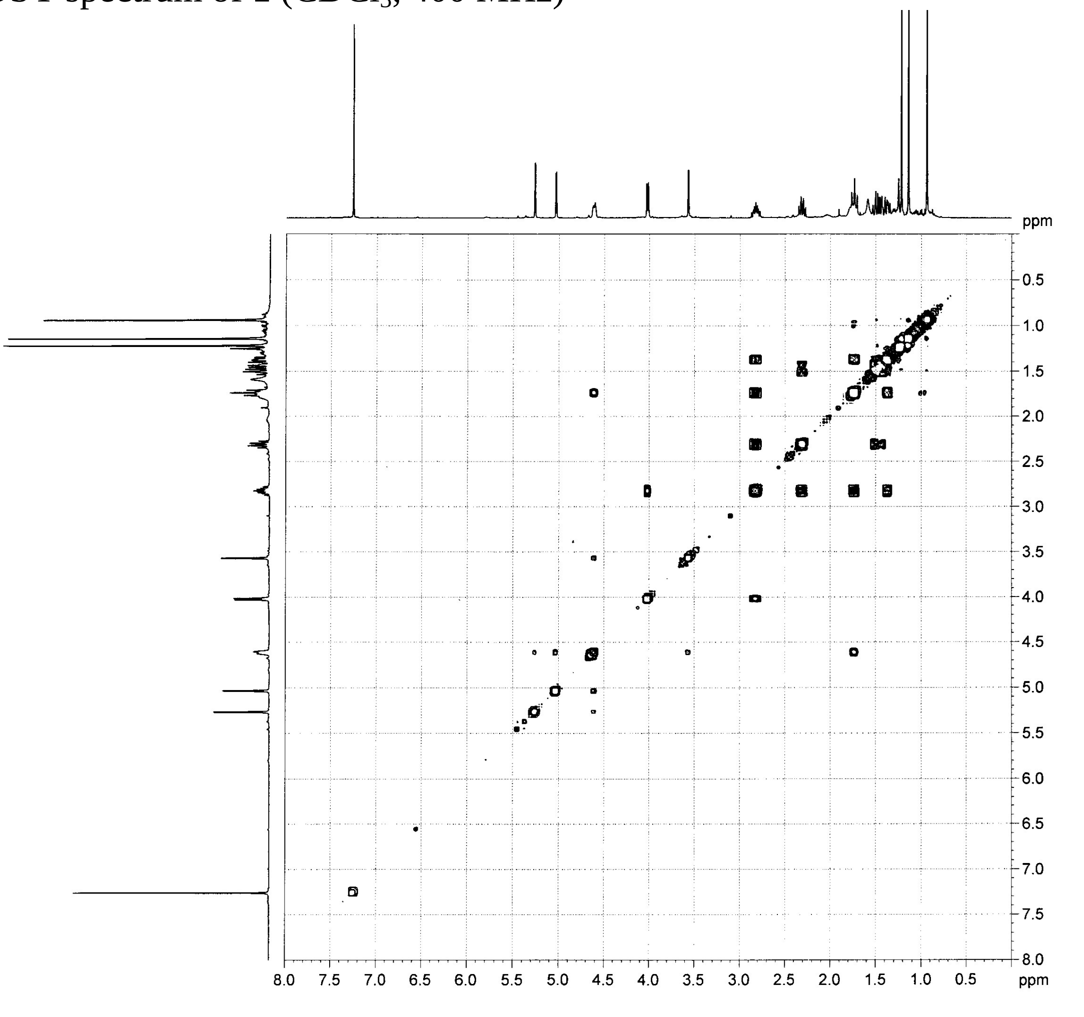

S5 NOESY spectrum of **1** ($CDCl_3$, 400 MHz)

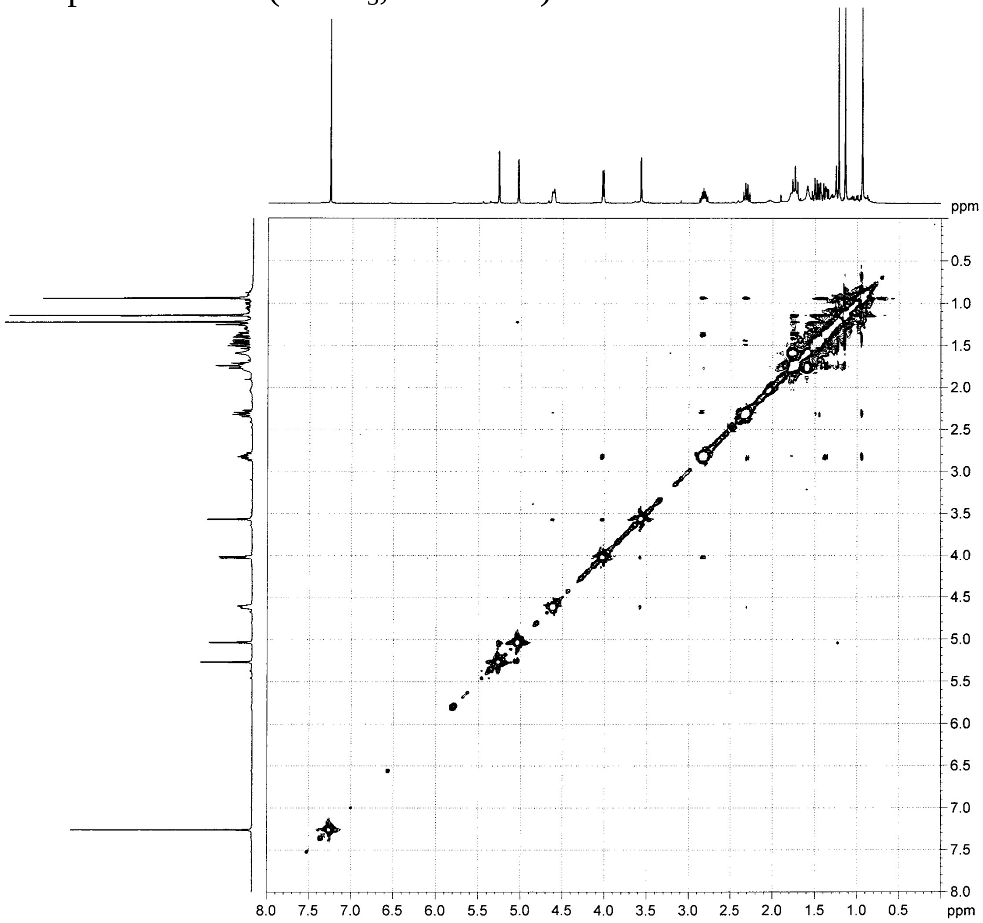

S6 HMQC spectrum of **1** ($CDCl_3$, 400 MHz)

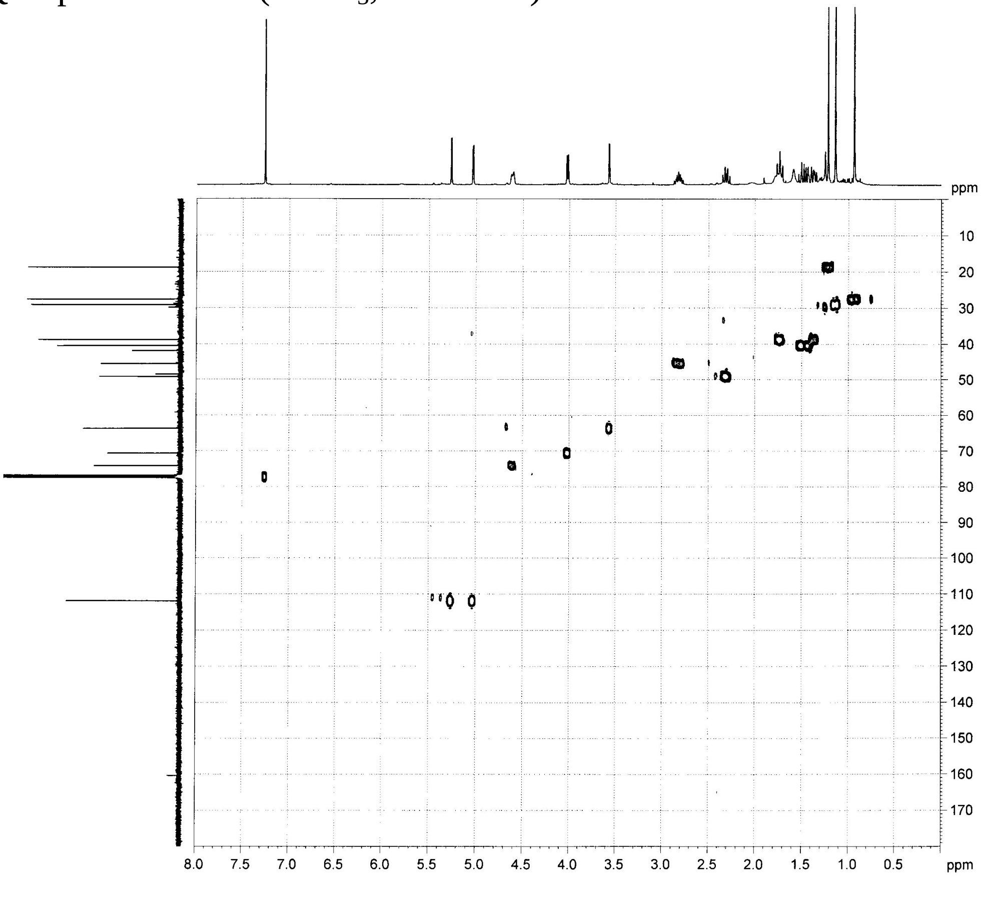

S7 HMBC spectrum of **1** ($CDCl_3$, 400 MHz)

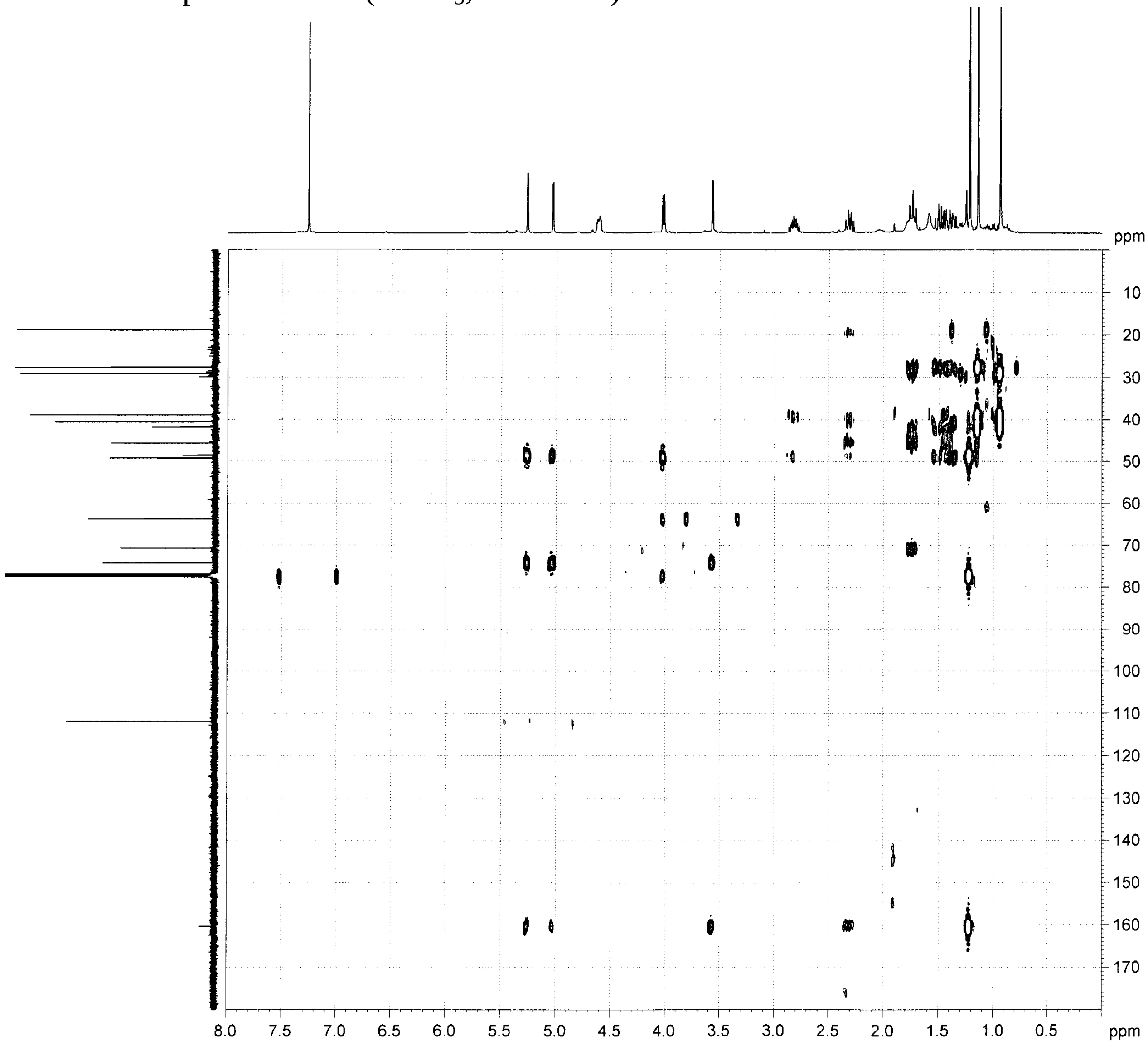

S8 ^{1}H NMR spectrum of **2** ($CDCl_3$, 400 MHz)

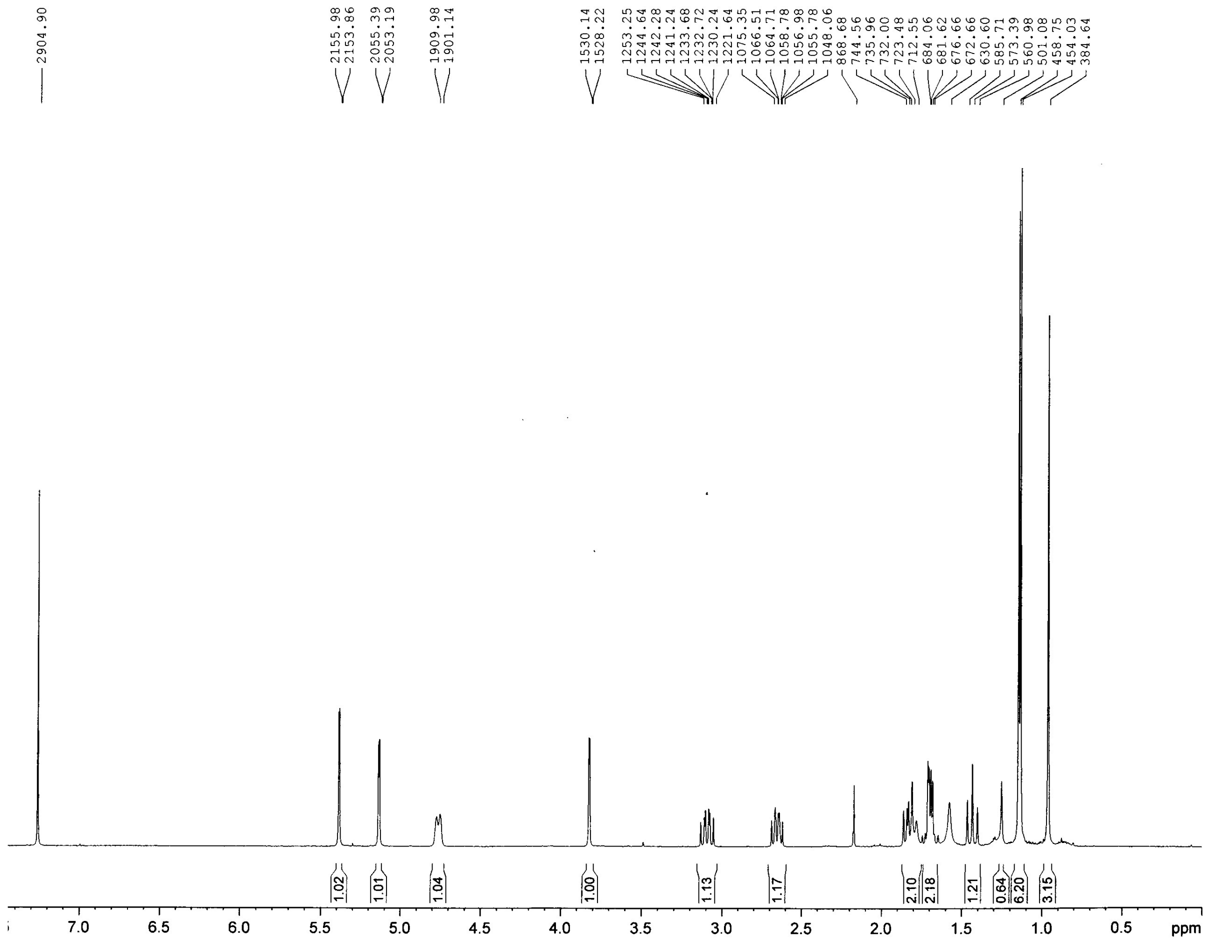

S9 ^{13}C NMR spectrum of **2** ($CDCl_3$, 100 MHz)

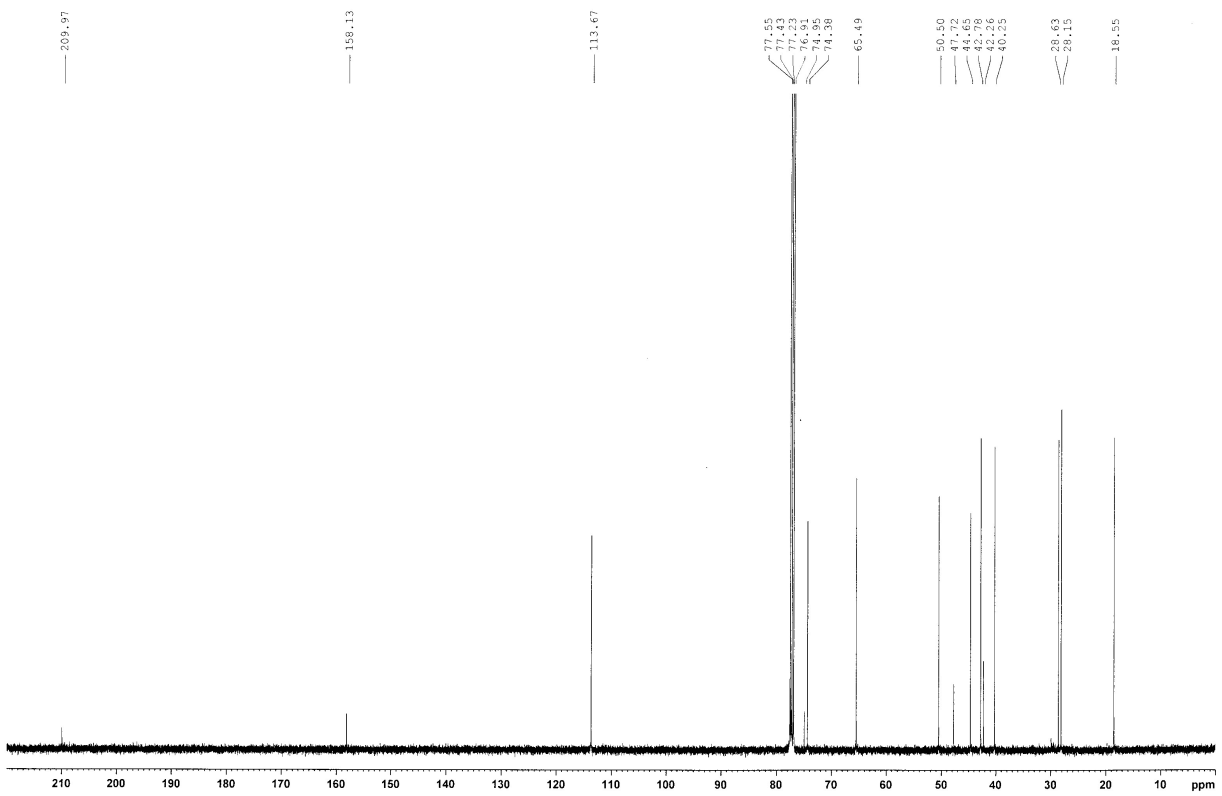

S10 DEPT135 spectrum of **2** ($CDCl_3$, 100 MHz)

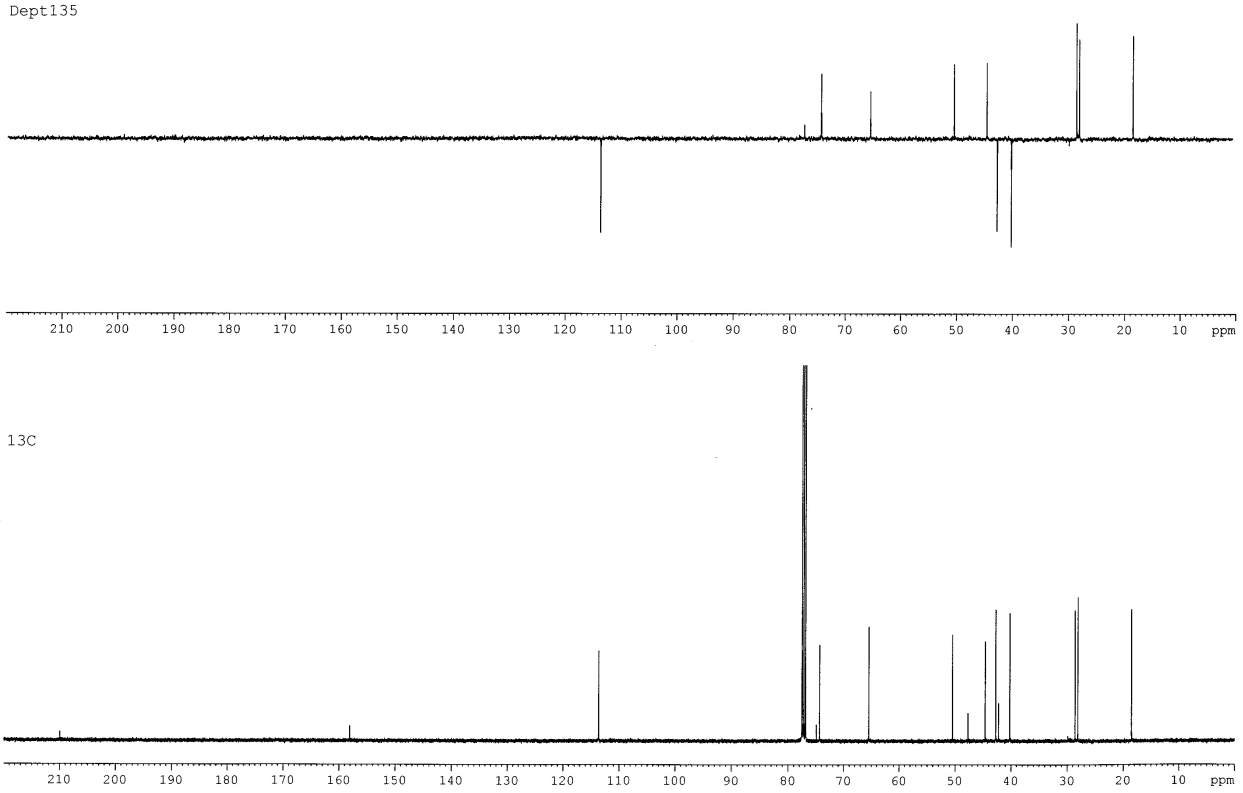

S11 COSY spectrum of **2** ($CDCl_3$, 400 MHz)

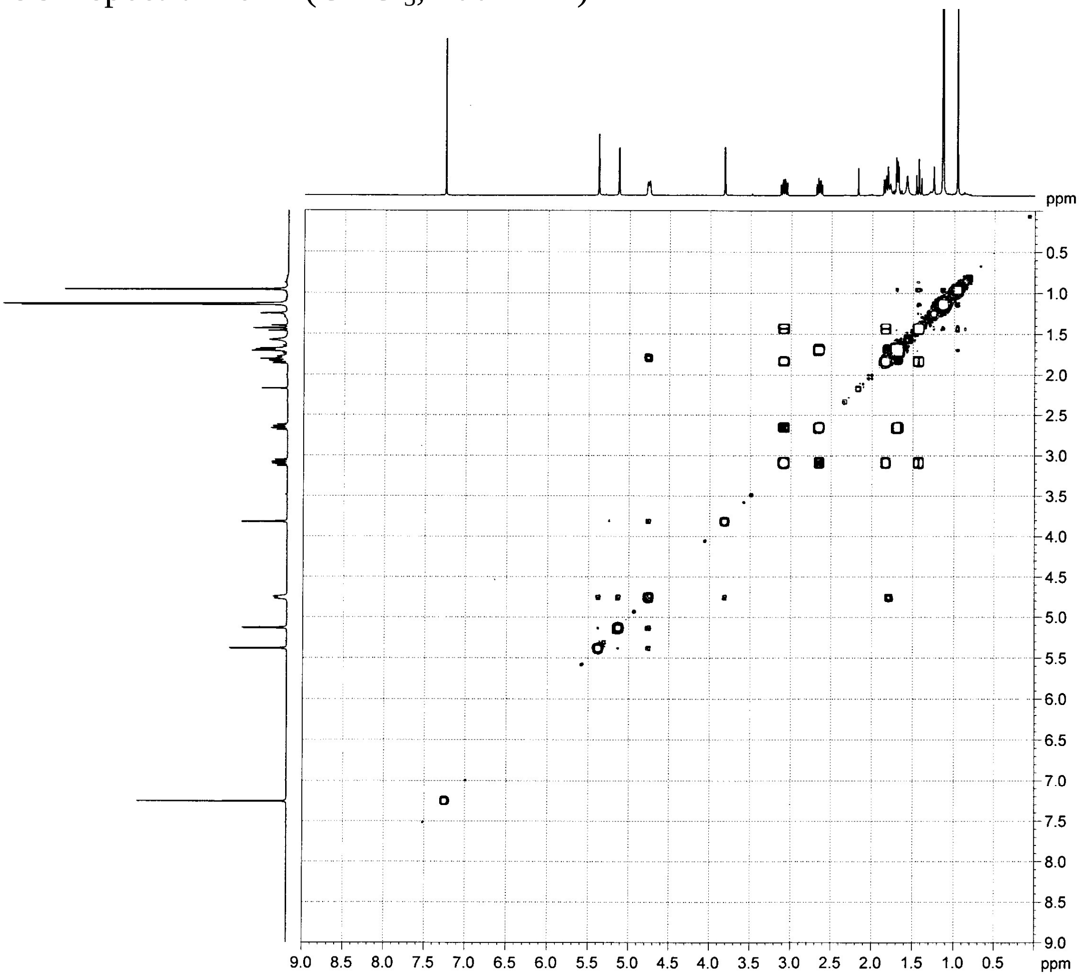

S12 NOESY spectrum of **2** ($CDCl_3$, 400 MHz)

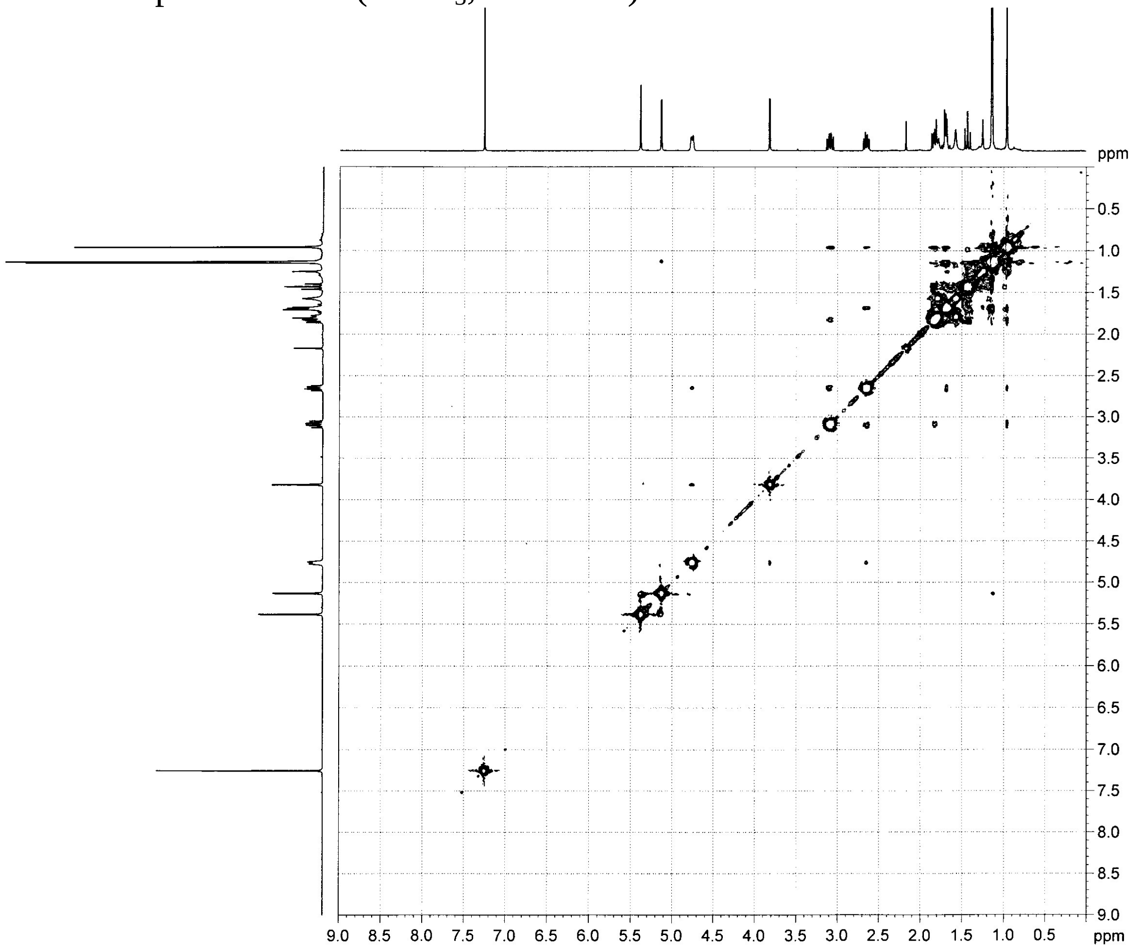

S13 HMQC spectrum of **2** ($CDCl_3$, 400 MHz)

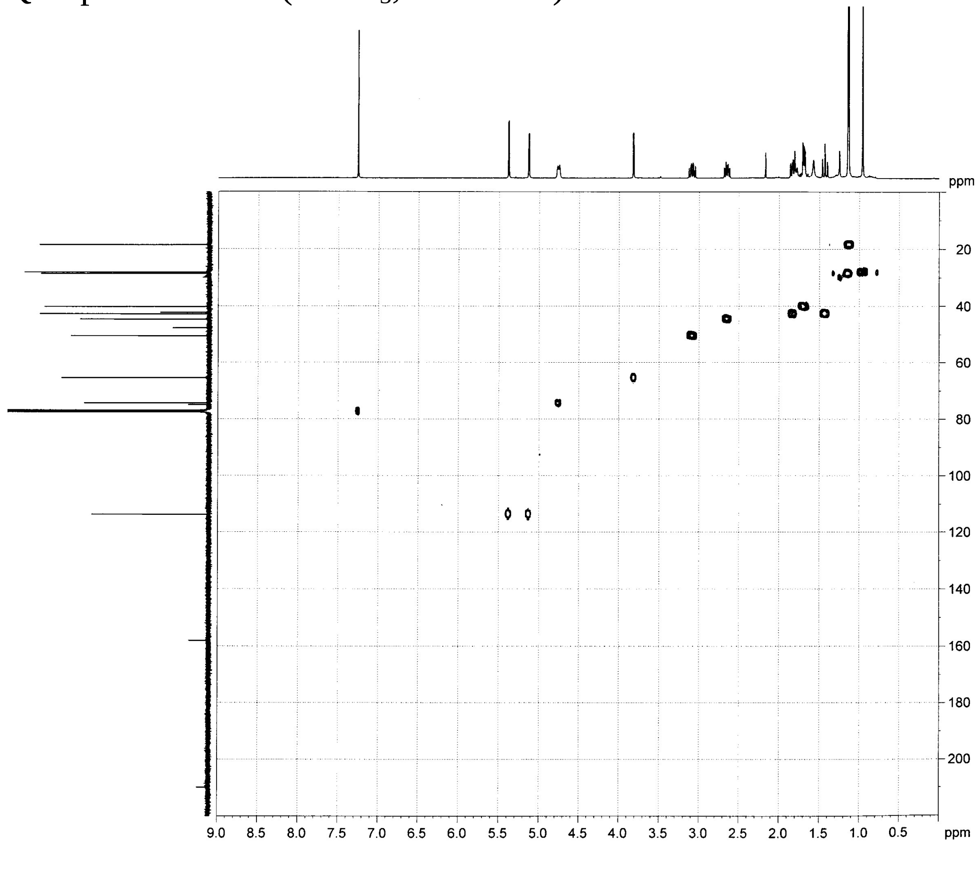

S14 HMBC spectrum of **2** ($CDCl_3$, 400 MHz)

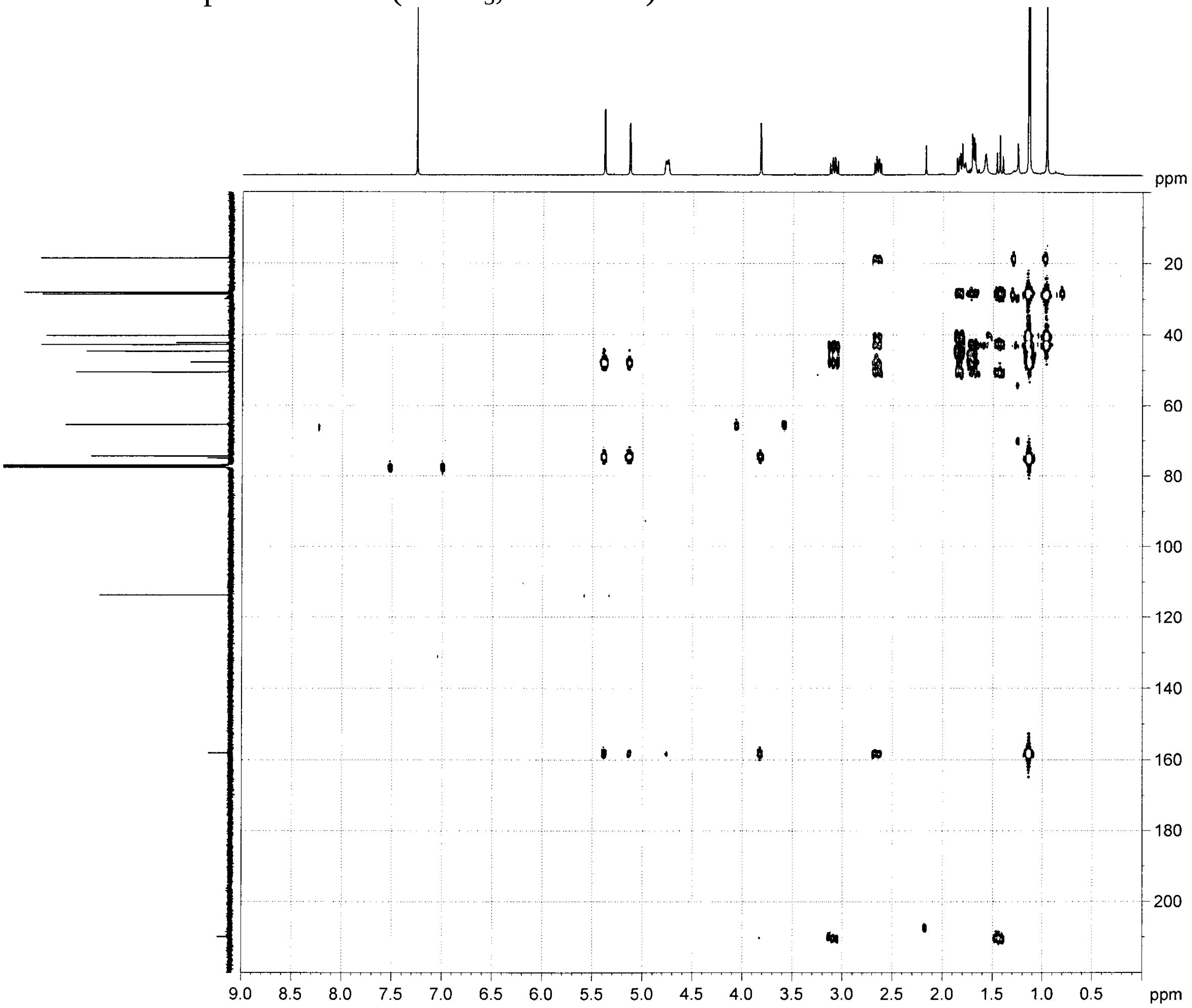

Supplement: Supplementary file 1 — Supplementary material 1 (PDF 3174 kb) [file 13659_2016_105_MOESM1_ESM.pdf]
